# Supplementary material for: Substituted cysteine modification and protection indicates selective interactions of the anesthetic photolabel pTFD-di-iPr-BnOH with α+/β– and α+/γ– transmembrane subunit interfaces of synaptic GABAA receptors
Source: PLoS One. 2025 Nov 6;20(11):e0336606. doi: 10.1371/journal.pone.0336606 (PMC12591484; doi:10.1371/journal.pone.0336606)
Supplement: S1 Fig — (PDF) [file pone.0336606.s001.pdf]

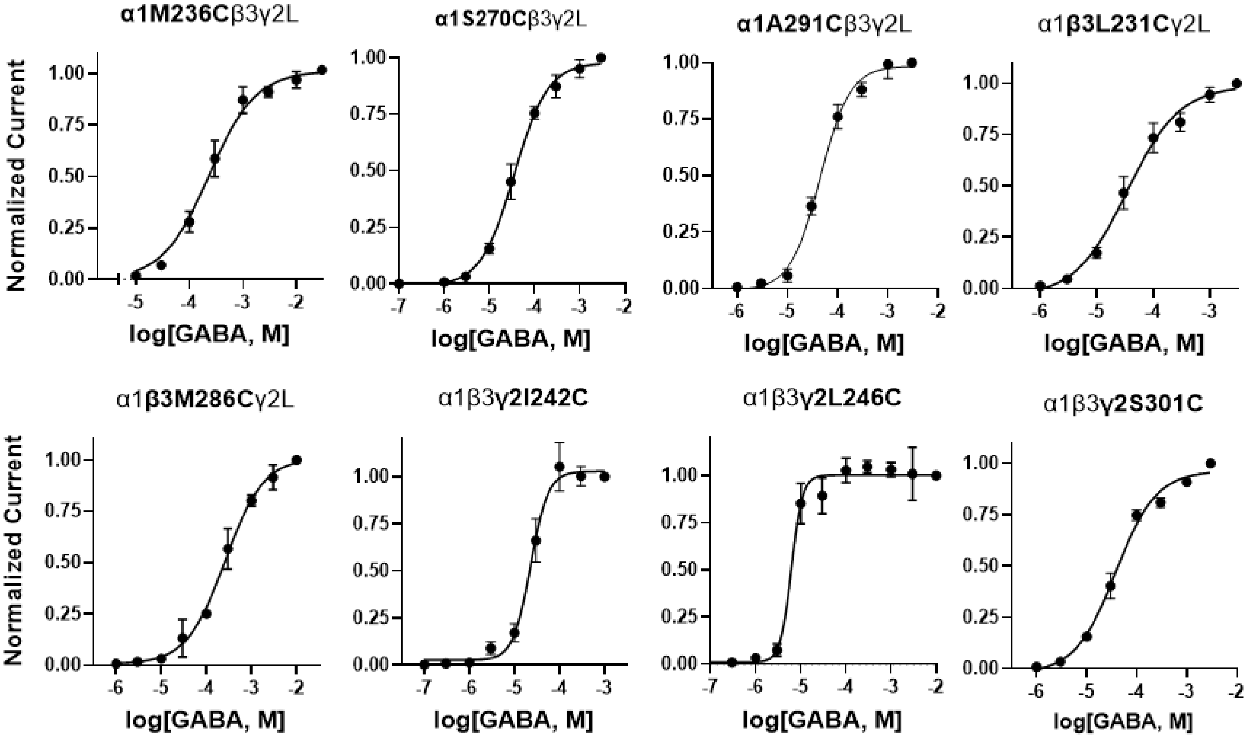


**Figure S1: GABA Concentration Responses of Cysteine Substituted $GABA_A$ Receptors.** Each panel depicts normalized peak current responses to varying GABA concentrations in voltage-clamped oocytes (mean ± SD; n = 5) expressing α1β3γ2L $GABA_A$ receptors with a single cysteine mutation (labeled above panels). Peak currents were normalized to peak currents stimulated with maximal GABA (3 mM to 30 mM) in the same oocyte. Pooled data were analyzed with non-linear least squares fits to logistic functions (Eq. 1) with variable slopes. GABA $EC_{50}$s from fits are reported in Table 1, along with the $EC_{50}$ fitted to wild-type receptor results.
