# Supplementary material for: Substituted cysteine modification and protection indicates selective interactions of the anesthetic photolabel pTFD-di-iPr-BnOH with α+/β– and α+/γ– transmembrane subunit interfaces of synaptic GABAA receptors
Source: PLoS One. 2025 Nov 6;20(11):e0336606. doi: 10.1371/journal.pone.0336606 (PMC12591484; doi:10.1371/journal.pone.0336606)
Supplement: S2 Fig — (PDF) [file pone.0336606.s002.pdf]

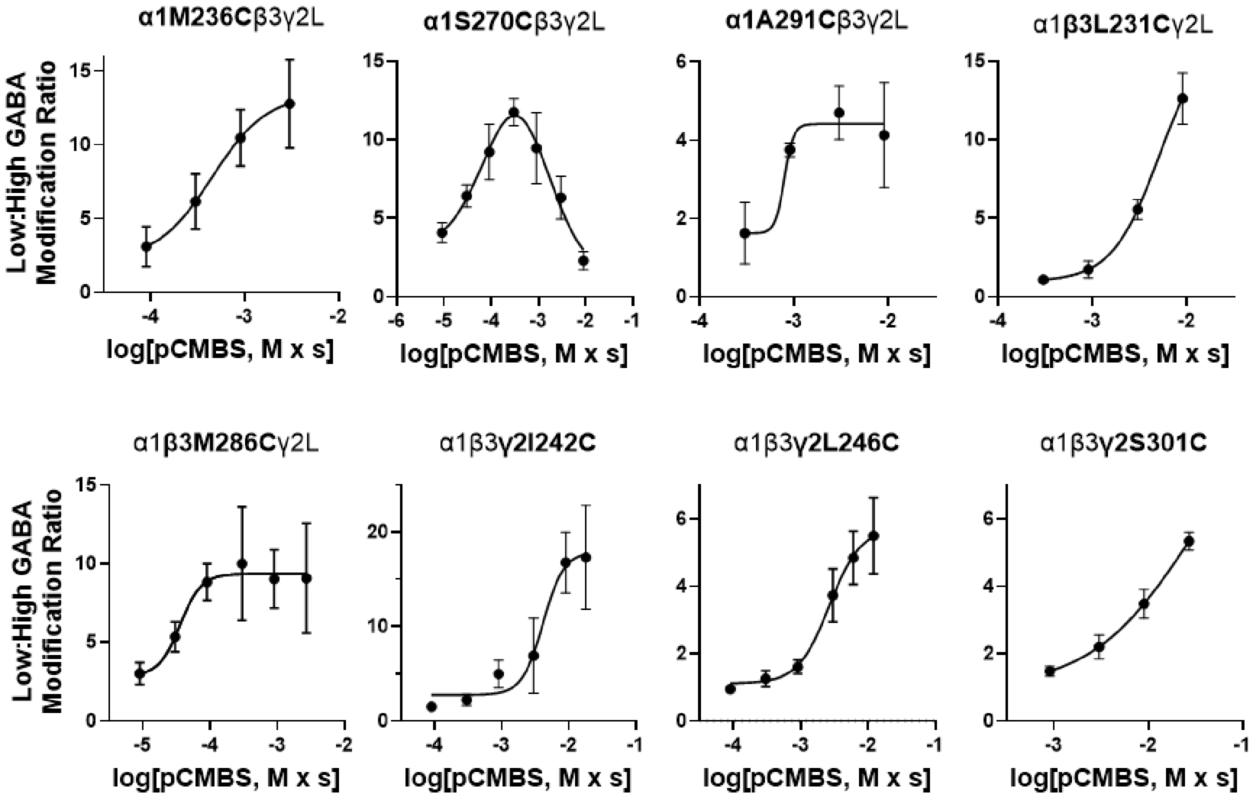

Figure S2: Functional Effects of Varying pCMBS Exposures in Cysteine Substituted Mutant α1β3γ2L GABA$_A$ Receptors. Each panel displays Low/High GABA Modification Ratios (mean ± SD; n = 5) for oocytes expressing a cysteine substituted mutant α1β3γ2L GABA$_A$ receptor (labels above panels) when exposed to maximal GABA plus the covalent sulfhydryl modifier pCMBS at varying concentrations for varying times. Exposures were calculated as M x s with a maximal exposure of 100 μM pCMBS for 90s (9000 μM x s). All mutants showed increasing GABA sensitivity (higher Low/High GABA Modification Ratios) with increasing pCMBS exposures. The α1S270Cβ3γ2L receptors showed a biphasic response to increasing pCMBS exposure. Lines through data points are mono-phasic or bi-phasic logistic non-linear least squares fits. Results were used in selection of control modification conditions for protection studies, reported in Table 1. pCMBS = para-chloromercuribenzenesulfonate.
